# Protective Effects of Lactic Acid Bacteria Against TLR4 Induced Inflammatory Response in Hepatoma HepG2 Cells Through Modulation of Toll-Like Receptor Negative Regulators of Mitogen-Activated Protein Kinase and NF-κB Signaling

**DOI:** 10.3389/fimmu.2018.01537

**Published:** 2018-07-04

**Authors:** Paulraj Kanmani, Hojun Kim

**Affiliations:** Department of Korean Medicine, Dongguk University, Goyang, South Korea

**Keywords:** probiotics, immunoregulatory activity, hepatic steatosis, hepatic inflammation, lipopolysaccharide, hepatoma cells

## Abstract

The beneficial effects of probiotics in several liver diseases have been investigated in both animal and clinical models; however, the precise mechanisms responsible for their effects have not yet been elucidated. Gut transmitted endotoxins such as LPS have been shown to play critical roles in hepatic inflammation and injury. Therefore, in this study, we investigated the beneficial role of selected lactic acid bacteria (LABs) on reduction of hepatic steatosis (HS) and attenuation of LPS induced inflammatory response *in vitro*. Total cellular fluid (TCF) of LABs treatment reduced HS by decreasing the amount of lipid accumulation *in vitro*. Additionally, HepG2 cells exposed to LPS showed increased expression of exacerbated inflammatory cytokines, such as IL-6, CXCL8, CCL2, and TNF-α, but these effects were counteracted when cells were treated with TCF of LABs prior to LPS challenge. Moreover, TCF of LABs was able to modulate mRNA levels of TLR negative regulators and protein levels of p38 MAPK and p65 NF-κB transcription factors. However, these modulations were differed remarkably between both free fatty acid treated and untreated HepG2 cells. Heat-killed LABs were also indirectly suppressed THP-1 cells to produce higher level of IL-10, TLR4, and lower at genes level of TGF-β, IL-1β, and IL-6, and at protein level of TNF-α in response to LPS. Taken together, our findings indicate that selected LABs exhibit profound immunoregulatory effects on liver cells via modulation of TLR negative regulators of the MAPK and NF-κB pathways.

## Introduction

Inflammation plays a pivotal role in disease and health, and is a prime immune response triggered by injury to living tissues. The inflammatory response is a protective mechanism that evolved in living things to protect against injury, infection, trauma, and noxious stimuli (1). The purpose of this process is to eliminate injurious agents or intruders and to clear the components of damaged tissues. Several factors can induce inflammation, however, microbes and their cellular components are considered to be the most common cause of inflammatory response in the host (2). Lipopolysaccharide (LPS) is a major cellular component of Gram-negative bacteria, which is able to induce pro-inflammatory pathways leading to an inflammation and progression of several disease, including alcoholic and nonalcoholic liver diseases (3–5). During the gut barrier disruption, LPS migrates from the intestinal lumen to other extra-intestinal body organs including liver *via* the portal vein, where it activates hepatic and extra hepatic macrophages to produce reactive oxygen species and pro-inflammatory cytokines/chemokines, such as IL-1, IL-6, IL-8, and TNF-α, resulting in liver inflammation (3, 5).

Toll-like receptors (TLRs) are the type of pattern-recognition receptors that helps host to detect invading pathogens and their related infections by recognizing pathogen associated molecular patterns (PAMPs) (5, 6). The functions of TLRs are not only in the regulation of innate and adaptive immune responses, but also plays role in the noninfectious inflammatory processes of the liver, lung, and cardiovascular system (7–9). TLR4 act as a receptor for LPS. The majority of human cell types, including hepatocyte cells can express TLRs. The expression of TLRs in hepatic cells could play role in the progression of diseases and local immunity of liver. Upon recognition of LPS by TLR4 mediates inflammatory signaling through the series recruitment of signaling proteins such as myeloid differentiation factor 88 (MyD88), interleukin-1 receptor associated kinase (IRAK), and TNF receptor associated factor 6 resulting in activation of mitogen-activated protein kinase (MAPKs) and nuclear factor kappa B (NF-κB) pathways (5, 7, 10). TLR4-mediated activation of MAPKs and NF-κB pathways induce an inflammatory state by producing an array of exaggerated inflammatory cytokines or mediators. Many studies showed the evidence for the pathogenic role of TLR4/LPS signaling in the progression of alcoholic and nonalcoholic fatty liver diseases [ALD, non-alcoholic fatty liver disease (NAFLD)] and hepatocellular carcinoma (3, 11, 12).

Human gut microflora play several beneficial roles in the host including maintenance of host immune homeostasis (13). Controlling of gut microflora reduced the translocation of endotoxins (LPS) and other bacterial derived toxic components such as phenol, ethanol, and indoles to the liver, which is in turn to reduce the liver injury by decreasing these components induced inflammatory cytokines production *via* downregulation of NF-κB pathway (14). Probiotics modulate the gut bacterial community, alter the intestinal lumen, and favor an anti-inflammatory milieu, which results in improvement of gut barrier integrity and reduction of bacterial and their cellular components translocation, leading to liver protection (5). Several clinical and experimental studies have reported the possible beneficial role of probiotic bacteria in the control and prevention of inflammatory and liver diseases (15–18). The administration of *L. rhamnosus* GG decreased alcohol induced hepatic steatosis (HS) and liver injury in an *in vivo* mouse model (19). Another *in vivo* study showed that *L. rhamnosus* GG was able to protect mice against alcohol induced hepatic inflammation and liver injury by reducing production of hepatic TNFα *via* inhibition of endotoxin-mediated TLR4 activation (20). Most of the studies evaluated the probiotic effects *in vivo*; however, there have been limited attempts with aim to analyze the beneficial role of probiotics *in vitro*, and no clear mechanisms for the beneficial actions of probiotics have been identified to date. Moreover, the beneficial effects of probiotics are strains-specific and their functional properties, therefore, the selection of potent immune active probiotic strains is very important to achieve target therapeutic effect. The aim of this study, therefore, was to examine whether probiotics treatment would reduce lipid accumulation and attenuate exacerbated inflammatory response induced by bacterial LPS in hepatoma HepG2 cells, and attempted to unveil the underlying molecular mechanisms involved in the immunoregulatory activity of probiotic strains. The accumulation lipid, expression of inflammatory and anti-inflammatory cytokines, TLR negative regulators, and phosphorylation of MAPKs and NF-κB proteins were examined.

## Materials and Methods

### Cell Culture and Induction of HS

In this study, we used human hepatoma HepG2 cells that were maintained in DMEM medium, and then cultured in DMEM/High glucose medium (Gibco™) supplemented with 10% FBS and 1% penicillin/streptomycin at 37°C, in a humidified atmosphere of 5% CO_2_. During the experiment, the media was changed every alternative day for 5–6 days, and cells between 20 and 40 passages were used. To induce HS, HepG2 cells were plated at 5 × 10^4^ cells/ml in 12-well type I collagen coated plate (SPL Life Sciences Co. Ltd., Gyeonggi-do, South Korea) and incubated at 37°C for 3–4 days. The confluent cells were then incubated with free fatty acid (FFA) medium for 24 h. The FFA medium was DMEM that contained 10% FBS, 1% penicillin/streptomycin, 1% bovine serum albumin, and 1 mM FFA [2:1 oleic acid (0.66 mM) and palmitic acid (0.33 mM) prepared in 100% isopropanol]. Fat accumulated cells were then washed with distilled phosphate buffered saline (DPBS) prior to further use.

In addition, we also used human monocytic cells (THP-1) that were cultured in RPMI-1640 medium (Gyeongsagbuk-do, South Korea) supplemented with 1% FBS, 1% penicillin/streptomycin, and 0.05 mM mercaptoethanol at 37°C for 5–6 days. The cells were then incubated with differentiation medium [RPMI 1640 media with 50 ng/ml of PMA (phorbol 12-myrisate 13 acetate)] to differentiate into macrophage like cells. After 48 h, the differentiation medium was replaced with fresh RPMI medium, and the samples were incubated at 37°C for 24 h. The differentiated cells were then co-cultured with HepG2 cells and preparation of total cellular fluid (TCF) of LAB strains.

### Bacterial Strains and Preparation of TCF

*Lactobacillus plantarum* DU1, *L. farciminis, Weissella cibaria* DU1, and *L. pentosus* were isolated from fermented foods and characterized phenotypically and genotypically. The identified LAB strains were then cultured in MRS broth at 37°C for 19 h, centrifuged, washed with DPBS, and then suspended in Dulbecco’s modified Eagle’s Medium (DMEM, GIBCO, USA) at appropriate concentrations and kept at −4°C for further experimental usage. To prepare TCF, differentiated THP-1 cells (1 × 10^6^ cells/well) were cultured in 6-well cell culture plate and stimulated with LAB strains (5 × 10^7^ cells/well) for 24 h in RPMI media. Following stimulation, the TCFs were collected to fresh tubes and centrifuged to remove cell debris, after which they were filter sterilized and then stored −4°C until further stimulation. The cytotoxicity of the LAB strains and TCF was determined using a cell viability, proliferation and cytotoxicity assay kit (EZ-CYTOX, DOGEN Bio co. Ltd.) using HepG2 cells.

### Oil Red O Staining

To determine the effect of LAB strains on reduction of lipid accumulation, HepG2 cells were seeded (3 × 10^4^ cells/ml) in 12-well plate and incubated at 37°C for 3 days. The cells were then treated separately with TCF (25 µl and 50 µl/ml) of lactic acid bacteria (LABs) and LPS (1 µg/ml) for 48 h, after treatment with 1 mM FFA for 24 h. The treated cells were subsequently washed with DPBS and fixed with 10% formalin for 5 min at room temperature (RT). Next, the formalin was replaced with fresh 10% formalin and cells were incubated at RT for 1 h, and then washed with 60% isopropanol. The plate was then allowed to dry completely, after which it was stained with 0.5% Oil red O solution (3:2 ratio of ORO:water) for 10 min at RT. Thereafter, the stained cells were washed with deionized water (DW) four times and observed under a fluorescence microscope (Leica DMI 6000B, Wetzlar, Germany). Dried plates were then incubated with 100% isopropanol (1 ml/well) for 5 min and read at 520 nm using a microplate reader (SpectraMax Plus 384, San Jose, CA, USA) to quantify the amount of lipids that had accumulated in the cells.

### Anti-Inflammatory Activity of TCF of LAB Strains *In Vitro*

HepG2 cells were cultured (3 × 10^4^ cells/ml) in 12-well type I collagen coated plate (SPL Life Sciences Co., Ltd., Gyeonggi-do, South Korea) at 37°C under 5% CO_2_, and followed by treated with FFA for 24 h. Then, both FFA treated and not treated cells were pre-stimulated with TCF (50 µl) of LAB strains for 48 h. After washing with DMEM medium, the both cells were post-stimulated with 1 µg/ml of lipopolysaccharide (LPS from *E. coli* O55.B5, Sigma, USA) for 3 and 12 h. The expression of pro-inflammatory cytokines/chemokines, such as IL-6, CXCL8, CCL2, and TNF-α was then analyzed by RT-PCR as described below.

### Effect of LAB Strains on Reduction of Inflammatory Response in Co-Culture Model

HepG2 cells were seeded at 3.5 × 10^4^ cells/well in the upper chamber of transwell culture inserts [(transparent PTFE membrane coated collagen, 0.4 µm pore size) Transwell-COL, Corning Incorporated, New York, NY, USA], and incubated at 37°C under 5% CO_2_ for 5–6 days. This setup was then co-cultured with THP-1 cells that were taken in lower chamber. To analyze the anti-inflammatory activity of LAB strains, the apical monolayer of HepG2 cells (TEER value 486 Ω cm^2^) were stimulated with heat-killed LABs strains. After 48 h, fresh medium with LPS (1 µg/ml) was added to the basolateral side and the samples were incubated at 37°C. After 12 h of stimulation, the cellular fluid from both the apical and basolateral sides was collected and stored at −4°C until used to estimate cytokine production at the protein level. The RNA from THP-1 cells was extracted and subjected to RT-PCR to analyze the expressions of IL-10, TGF-β, IL-6, IL-1β, TLR2, and TLR4.

### Enzyme Linked Immunosorbent Assay (ELISA)

The amount of TNF-α production at the protein level in the cell free supernatant (CFS) of both HepG2 and THP-1 cells was estimated using a Human TNF-α Quantikine ELISA kit (R&D System, MN, USA) according to the manufacturer’s instructions.

### Modulation of TLR Negative Regulators Expressions in HepG2 Cells

To analyze the expression of negative regulators, FFA treated and not-treated HepG2 cells were stimulated with TCF of different LAB strains, and then incubated at 37°C under 5% CO_2_ for 48 h. After three washes with DMEM medium, the cells were post-stimulated with 1 µg/ml of LPS for 3 and 12 h. The cells were then washed with DPBS, scraped by adding TRIzol reagent and stored at −4°C to extract the RNA. The mRNA levels of TLR negative regulators such as SIGIRR, Tollip, A20, and IRAKM1 were analyzed by RT-PCR using the primers shown in Table 1.

**Table 1:** List of primers used in this study for RT-PCR.

Target gene	Primer sequences	Annealing temperature
β-actin	|F5-CAAGAGATGGCCACGGCTGCT-3 R5-TCCTTCTGCATCCTGTCGGCA-3	63°C
IL-6	F5-CATCCTCGACGGCATCTCAG-3 R5-GCTCTGTTGCCTGGTCCTC-3	63°C
CXCL8	F5-CTGGCCGTGGCTCTCTTG-3 R5-CCTTGGCAAAACTGCACCTT-3	63°C
CCL2	F5-CTCAGCCAGATGCAATCAATG-3 R5-AGATCACAG CTTCTTTGGGACAC-3	63°C
IL-1-β	F5-GTG GCA ATG AGG ATG ACT TGT TC-3 R5-TTG CTG TAG TGG TCG GAG-3?	60°C
IL-10	F5-TCA GGG TGG CGA CTC TAT-3 R5-TGG GCT TCT TC TAA ATC GTT C-3	60°C
TLR2	F5-GCA GAA GCG CTG GGG AAT GG-3? R5-GGA TGC CTA CTG GGT GGA GAA-3	60°C
TLR4	F5-GGT GGA AGT TGA ACG AAT GG-3 R5-CCA GCA AGA AGC ATCAGG TG-3	60°C
TGF-β	F5-GCT GCT GTG GCT ACT GGT GC-3 R5-CAT AGA TTT CGT TGT GGG TTT C-3	60°C
A20	F5-CTG CCC AGG AAT GCT ACA GAT AC-3 R5-GTG GAA CAG CTC GGA TTT CAG-3	63°C
IRAKM-1	F5-AGC TGC GGG ATC TCC TTA GAG-3 R5-ACC GGC CTG CCA AAC AG-3	63°C
Tollip	F5-CAG GCG TGG ACT CTT TCT ATC TC-3 R5-GAC TCC GGG ATG GTG ATG TG-3	63°C
SIGIRR	F5-TTC AGT CCA GTG GCT GAA AGA CGG-3 R5-ACC TCT GAC AGG TTG GCC TTG AC-3	63°C

### Western Blot

To analyze the phosphorylation of p38 MAPK and p65 NF-κB proteins, HepG2 cells were cultured (1.8 × 10^5^ cells/dish) in dishes (60 mm) at 37°C under a humidified atmosphere of 5% CO_2_ for 5–6 days. The cells were then stimulated with TCF of *L. plantarum* DU1, *L. farciminis, W. cibaria* DU1, *L. pentosus*, and *L. sakei* at 37°C under 5% CO_2_ for 48 h. Next, cells were washed with medium and stimulated with LPS (1 µg/ml) for 0, 10, 30, 60, and 120 min. After washing with DPBS, 200 µl of CellLytic M cell lysis reagent (Sigma-Aldrich, St. Louis, MO, USA) was added to the dishes and kept on ice for 5 min to lyse the cells. The lysed cells were subsequently scraped off and transferred to fresh Eppendorf tubes, then stored at −70°C until blot analysis. The concentration of protein in the lysed sample was analyzed using a bicinchoninic acid assay kit (Thermo Scientific, Pierce, Rockford, IL, USA), after which the samples were heated at 95°C for 5 min, then loaded into SDS-polyacrylamide gels (10%). The separated proteins were transferred to a nitrocellulose membrane (Trans-Blot Turbo™, Hercules, CA, USA), after which the transferred membrane was cut at the desired part and incubated with blocking buffer prior to incubation with various antibodies.

The phosphorylation of p38 and degradation of p65 were evaluated by incubating membranes with MAPK Phospho-p38α (T180/Y182) antibody (p-p38α, Cat. #MAB8691, R&D Systems, MN, USA); NF-κB phospho-p65 (p-p65, Cat. #9242); and β-actin antibody (Cat. #4970) from Cell Signaling Technology (Beverly, MA, USA) overnight at RT. Goat anti-rabbit IgG-HRP polyclonal antibody (AbFrontier, Cat. #LFSA8002) was used as a secondary antibody. The optical protein bands were detected by adding a mixture (1:1 ratio) of Western blot detection solution A and B (SUPEX, Neonex Co., Ltd., Postech, South Korea), after which, we estimated the area of the densitogram peak using the Image J software (National Institute of Health, Bethesda, MD, USA).

### Quantitative Real-time Polymerase Chain Reaction (qRT-PCR)

Total RNA was extracted with TRIzol reagent (Invitrogen) according to the method described by Ishizuka et al. (21). The purity and quantity of RNA was analyzed by the Nanodrop method (Nanodrop Technologies, USA), after which cDNA was synthesized using a thermal cycler (BIORAD, Hercules, CA, USA). Next, RT-PCR was conducted using a 7300 real-time PCR system (Roche Applied Science, Indianapolis, ID, USA) with SYBR green and the primers listed in Table 1. The reactions were conducted in mixtures of 1 µl of cDNA and 19 µl of master mix that included SYBR green and forward and reverse primers (1 pmol/μl). Amplification was performed by subjecting the samples to 50°C for 5 min, followed by 95°C for 5 min and then 40 cycles of 95°C for 15 s, 60°C–63°C for 30 s, and 72°C for 30 s. β actin was used as an internal control.

### Statistical Analysis

Statistical analyses were performed using the SPSS software package (SPSS 12.0, SPSS Inc., Chicago, IL, USA). One-way analysis of variance was performed and the significance of each mean value was determined by Tukey’s multiple range tests. A *p* < 0.05 was considered to indicate statistical significance.

## Results

**Figure 1:**
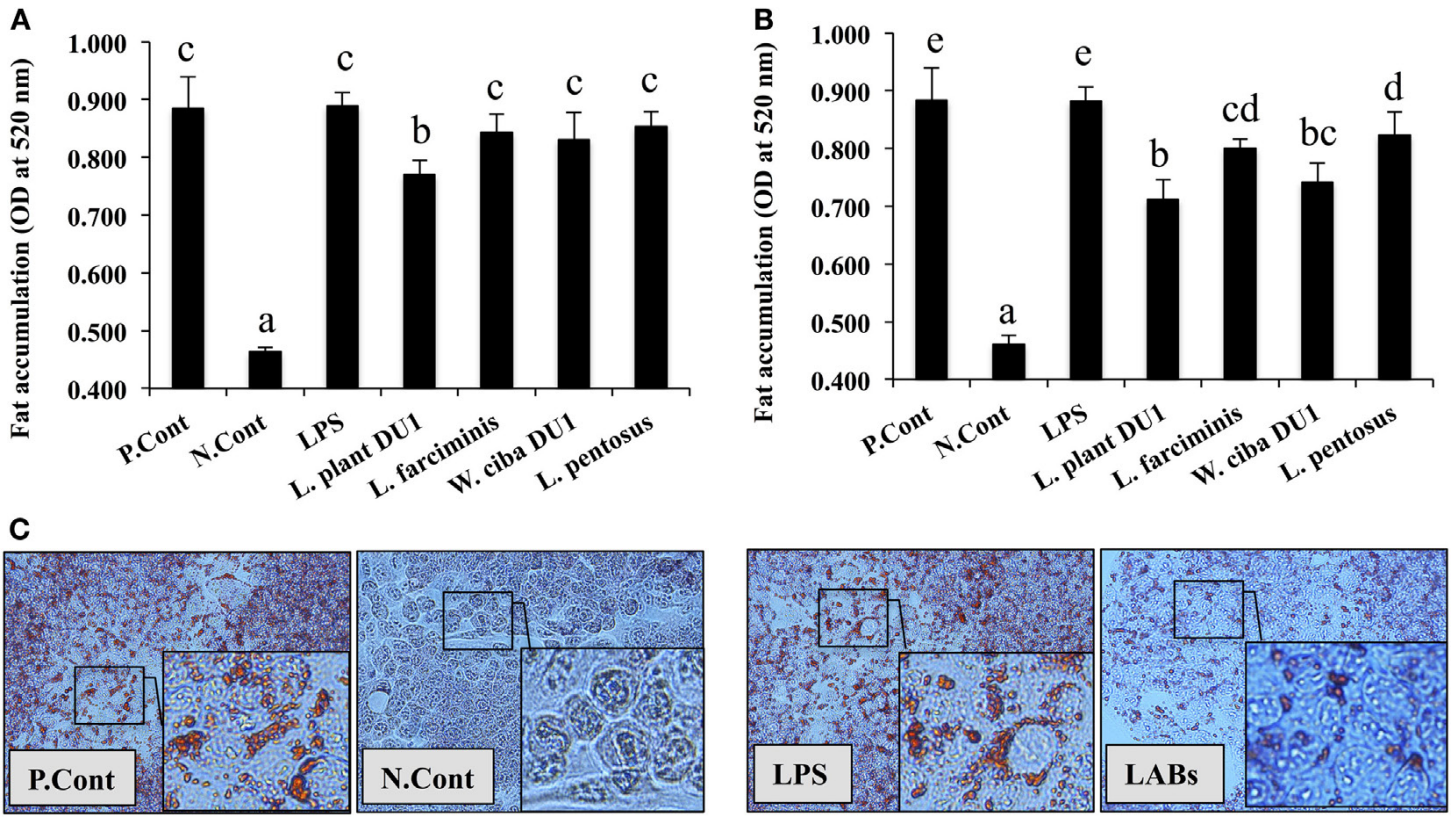
Total cellular fluid (TCF) of lactic acid bacteria (LAB) reduced accumulation of lipid in HepG2 cells. Confluence HepG2 cells were separately incubated with 25 µl **(A)** and 50 µl **(B)** of TCF of LAB strains and LPS (alone) for 48 h, after treated with free fatty acid (FFA) (1 mM) for 24 h. **(C)** Photographic pictures of HepG2 cells with lipid droplets. The amount of lipid accumulated in the treated cells was quantified by spectrophotometer at 520 nm, after ORO staining. The results presented as an average values (mean ± SD) of three independent experiments. Different superscript letters indicate significant differences at the 0.05 level. P. Cont and N. Cont are the short form of positive control (FFA alone treated) and negative control (normal cells).

Lactic acid bacteria, such as *L. plantarum* DU1, *L. farciminis, W. cibaria* DU1, and *L. pentosus* were isolated from fermented foods and characterized phenotypically and genotypically. The bacterial strains had no direct contact with liver cells *in vivo*; therefore, we treated HepG2 cells with TCF of THP-1 cells that had been stimulated with different LAB strains for 24 h. The cytotoxic effects of LABs and TCF were analyzed by incubation with HepG2 cells. There were no remarkable reductions observed in the percentage of cell growth. Next, we analyzed the effects of LAB TCF on reduction of fat accumulation in HepG2 cells. For this, cells were incubated with FFA for 24 h, prior to treated separately with TCF of LABs and LPS for 48 h. Cells treated with TCF of LABs showed profound reductions in the level of lipid accumulation (Figures 1A,B). However, 50 µl LAB TCF (Figure 1A) showed better reduction than 25 µl (Figure 1B). Moreover, the TCF of *L. plantarum* DU1 and *W. cibaria* DU1 were found to significantly reduce the accumulation of lipid in HepG2 cells by19.4 and 17.1%, respectively.

### Anti-Inflammatory Activity of LAB TCF in HepG2 Cells

**Figure 2:**
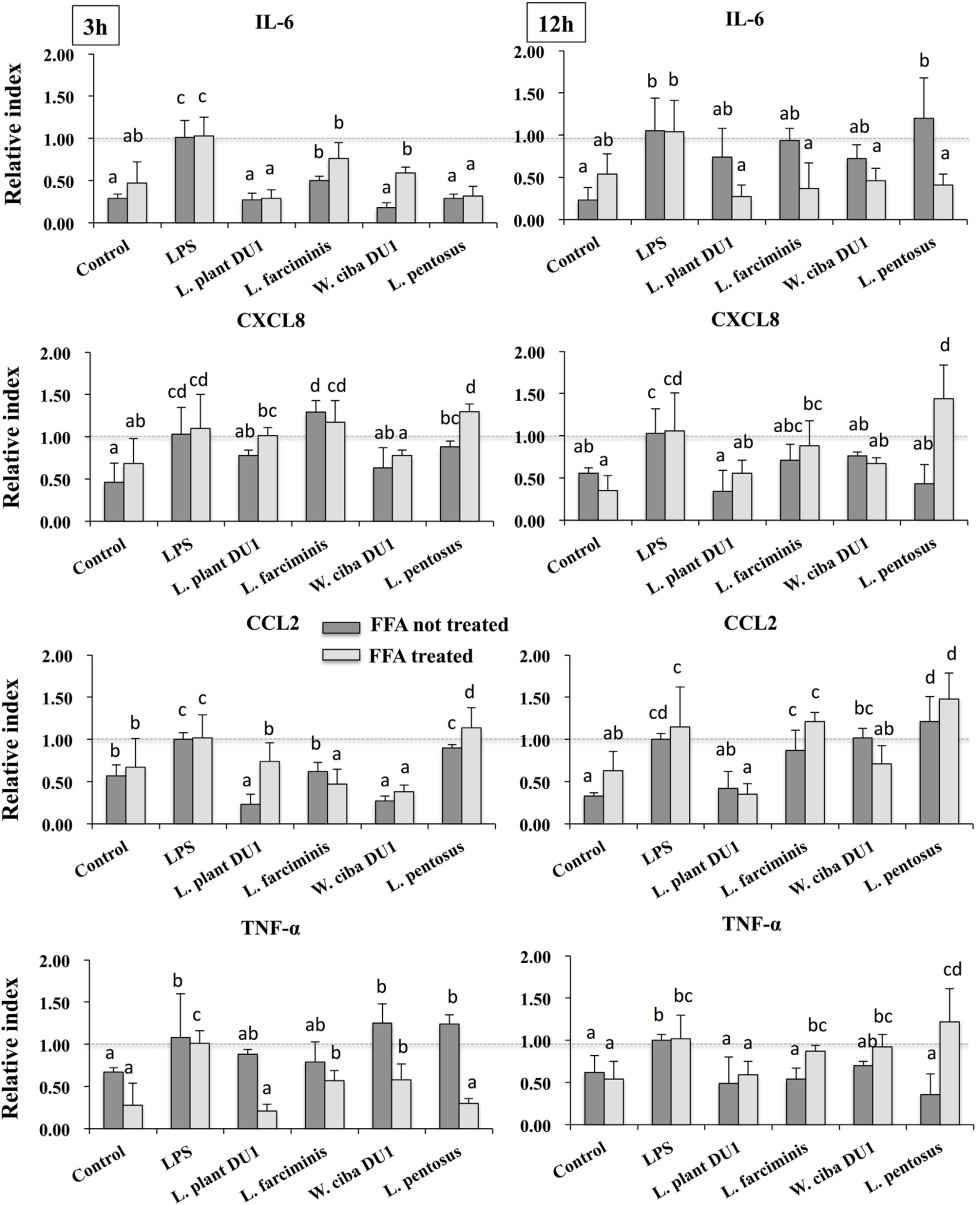
Total cellular fluid (TCF) of lactic acid bacteria (LAB) downregulated LPS induced pro-inflammatory cytokines/chemokine in HepG2 cells. Confluence HepG2 cells were pre-stimulated with LAB TCF (50 µl/ml) for 48 h, then stimulated with LPS for 3 and 12 h. The mRNA levels of IL-6, CXCL8, CCL2, and TNF-α were determined by RT-PCR. HepG2 cells either with medium or LPS was used as control. Three independent experiments were performed and the mean value was presented as anti-inflammatory effect of LABs. Different superscript letters indicate significant differences at the 0.05 levels.

We next analyzed the anti-inflammatory activity of LAB TCF in HepG2 cells. To accomplish this, FFA treated and not-treated HepG2 cells were stimulated with TCF of LABs for 48 h, then post-stimulated with LPS for 3 and 12 h. The results of RT-PCR showed that TCF of LABs were able to diminish the expression of inflammatory cytokines in time and cell-dependent manners (Figure 2). At 3 h, the TCF of all LABs except *L. pentosus* significantly decreased the mRNA level of IL-6 and CCL2 in response to LPS. Conversely, the expression of CXCL8 was not reduced by *L. farciminis* and *L. pentosus*, but other strains showed significant reductions in CXCL8 levels. The TCF of *L. plantarum* DU1 and *L. farciminis* suppressed the production of high levels of TNF-α by HepG2 cells not treated with FFA, while that of *W. cibaria* DU1 and *L. pentosus* failed to decrease its expression. In contrary, TCF of all LABs showed significant reductions in the levels of TNF-α in FFA-treated cells. However, at 12 h, cells stimulated with TCF of *L. plantarum* DU1, *L. farciminis*, and *W. cibaria* DU1 decreased the expression of IL-6, CXCL8, and TNF-α by HepG2 cells not treated with FFA, while *L. pentosus* was failed to suppress HepG2 cells to produce lower level of inflammatory cytokines except IL-6 (Figure 2). In contrast, all TCF except that of *L. plantarum* DU1 decreased the mRNA level of CCL2 compared to LPS in HepG2 cells not treated with FFA, but FFA treated cells responded differentially to TCF of LABs by showing different pattern of expression levels.

### Effect of LABs on Reduction of Inflammatory, Anti-Inflammatory Cytokines, and Receptor Expression in THP-1 Cells

**Figure 3:**
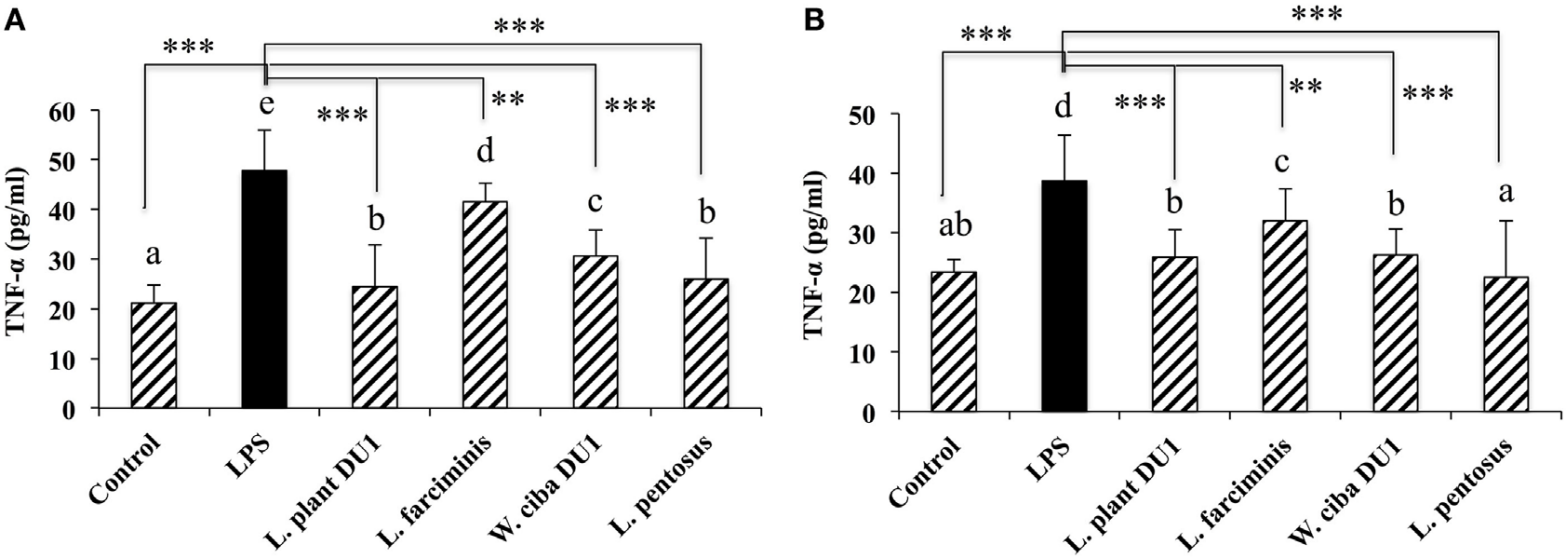
Lactic acid bacteria (LAB) strains reduce TNF-α production by THP-1 cells **(A)** and HepG2 cells **(B)**. In the co-culture setup, the apical side that contain HepG2 cells were stimulated with heat-killed LABs for 48 h, after which THP-1 cells taken in the basolateral side was treated with LPS for 12 h. The release of TNF-α in the both apical and basolateral compartment was determined by enzyme linked immunosorbent assay. Cells treated with either medium or LPS alone were used as controls. The results presented as an average values (mean ± SD) of three independent experiments. Different superscript letters indicate significant differences at the 0.05 level. Stars (** and ***) indicate significant differences at *p* < 0.005 and *p* < 0.0001 respectively.

**Figure 4:**
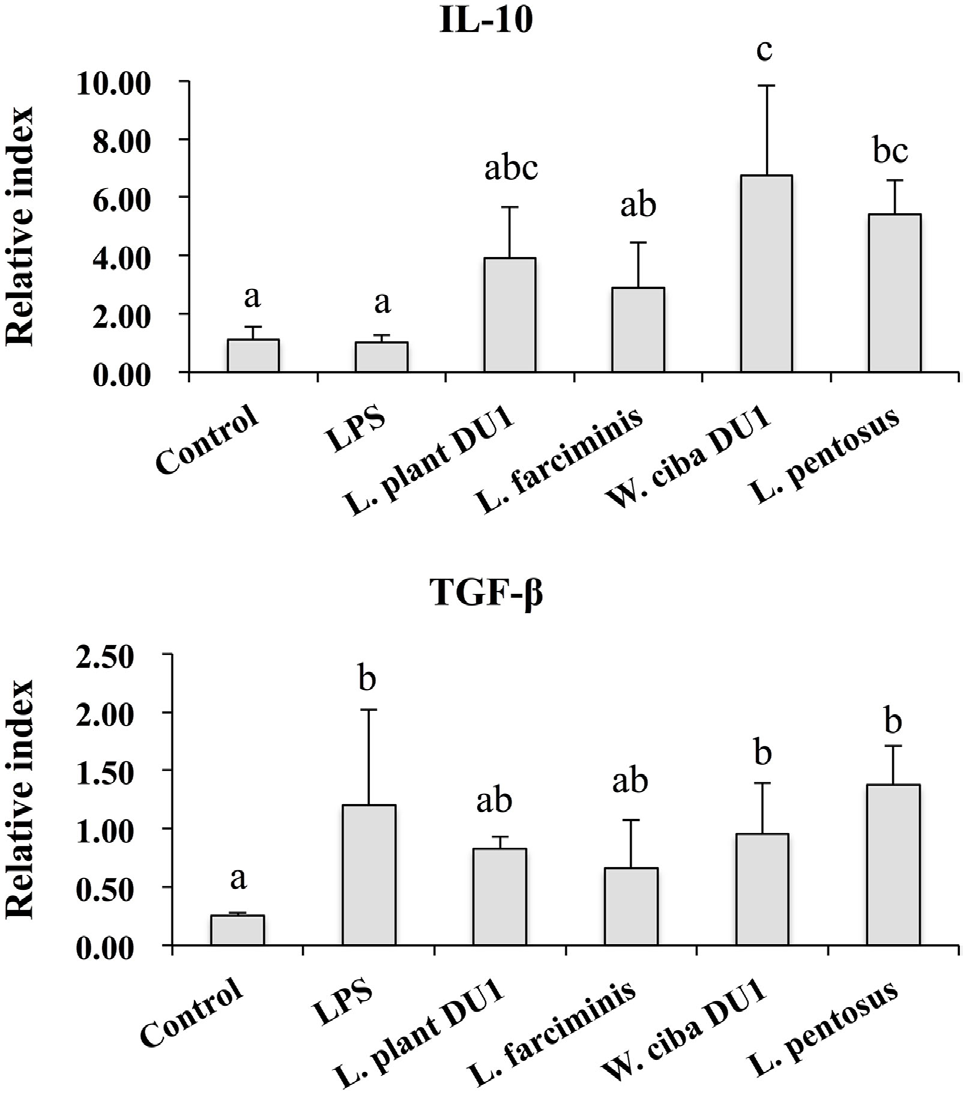
Lactic acid bacteria (LAB) strains altered the production of immunoregulatory and fibrogenic cytokine in THP-1 cells co-cultured with HepG2 cells. In the co-culture setup, the apical side that contain HepG2 cells were stimulated with heat-killed LABs for 48 h, after which THP-1 cells that taken in the basolateral side was treated with LPS for 12 h. The expressions of IL-10 and TGF-β at mRNA levels were determined by RT-PCR. Cells treated with either medium or LPS alone were used as controls. The results presented as an average values (mean ± SD) of three independent experiments. Different superscript letters indicate significant differences at the 0.05 level.

**Figure 5:**
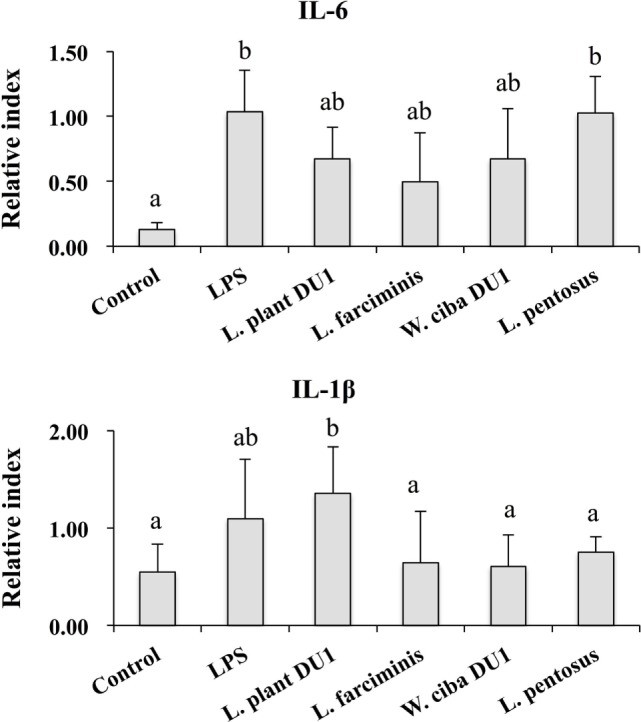
Lactic acid bacteria (LAB) strains reduced the expression of inflammatory cytokines in THP-1 cells co-cultured with HepG2 cells. In the co-culture setup, the apical side that contain HepG2 cells were stimulated with heat-killed LABs for 48 h, after which THP-1 cells that taken in the basolateral side was treated with LPS for 12 h. The expressions of IL-6 and IL-1-β were investigated by RT-PCR. Cells treated with either medium or LPS alone were used as controls. The results presented as an average values (mean ± SD) of three independent experiments. Different superscript letters indicate significant differences at the 0.05 level.

**Figure 6:**
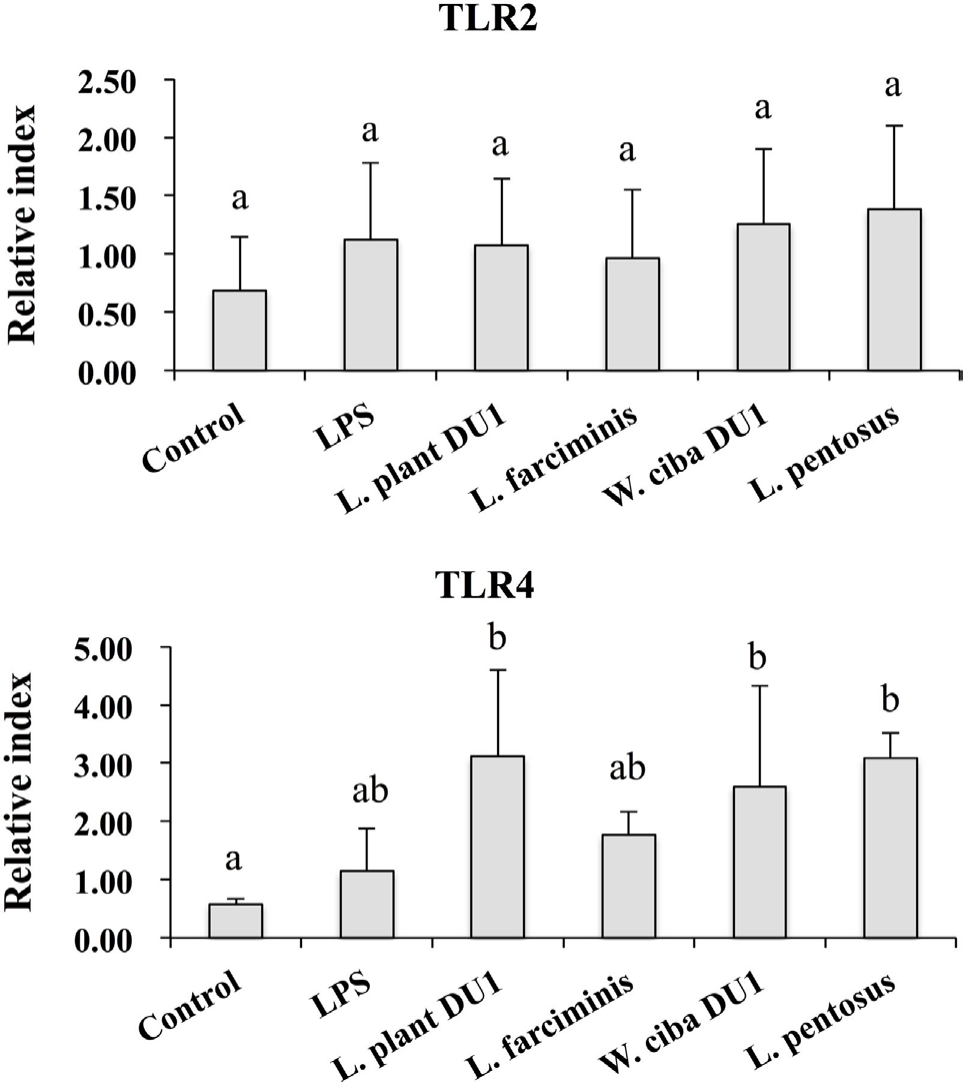
Effect of lactic acid bacteria (LABs) on expression of TLR2 and TLR4 on HepG2 sanitized THP-1 cells. In the co-culture setup, the apical side that contain HepG2 cells were stimulated with heat-killed LABs for 48 h, after which THP-1 cells that taken in the basolateral side was treated with LPS. After 12 h, the expressions of TLR2 and TLR4 were determined by RT-PCR. Cells treated with either medium or LPS alone were used as controls. The results presented as an average values (mean ± SD) of three independent experiments. Different superscript letters indicate significant differences at the 0.05 level.

We next analyzed whether LAB strains indirectly modulated the expression of inflammatory and anti-inflammatory cytokines in THP-1 cells. To accomplish this, we used a co-culture system in which HepG2 cells and THP-1 cells were cultured on the apical and basolateral side. After pre and post stimulation with heat-killed LABs and LPS, we examined the production of TNF-α at the protein level, as well as expression of IL-10, TGF-β, IL-6, IL-1β, TLR2, and TLR4 at the mRNA level in THP-1 cells. As shown in Figure 3, all LAB strains were able to reduce the production of TNF-α in both basolateral and apical sides. Cells stimulated with *L. plantarum* DU1, *W. cibaria* DU1, and *L. pentosus* showed better reduction than other LAB strains. Intriguingly, stimulation of cells with all LAB strains augmented the mRNA level of IL-10, while reduced the level of TGF-β, but not significantly in response to LPS (Figure 4). Among the strains, *W. cibaria* DU1 and *L. pentosus* stimulated the cells to produce significantly higher levels of IL-10 when compared with other LAB strains and LPS. Conversely, the level of TGF-β was markedly decreased when cells were stimulated with *L. plantarum* DU1 and *L. farciminis*; but *W. cibaria* DU1, *L. pentosus* exhibited relatively similar to level of LPS. In addition, we analyzed the expression of IL-6 and IL-1β in THP-1 cells (Figure 5). *L. plantarum* DU1, *W. cibaria* DU1, and *L. pentosus* decreased the mRNA levels of IL-6, but not remarkably as compared to LPS. *L. pentosus* was failed to show significant reduction in the level of IL-6. Furthermore, cells stimulated with LPS increased the expression of IL-1β compared to the control. LPS induced IL-1β was decreased by *L. farciminis, W. cibaria* DU1, and *L. pentosus*, while *L. plantarum* DU1 failed to suppress lower expression of IL-1β by THP-1 cells. Moreover, stimulation of cells with LABs altered the expression of TLR2, but not significantly, when compared to LPS (Figure 6). Conversely, all LABs except *L. farciminis* were significantly increased the mRNA level of LPS receptor TLR4 in THP-1 cells.

### Immunomodulatory Activity of TCF of LABs in HepG2 Cells

**Figure 7:**
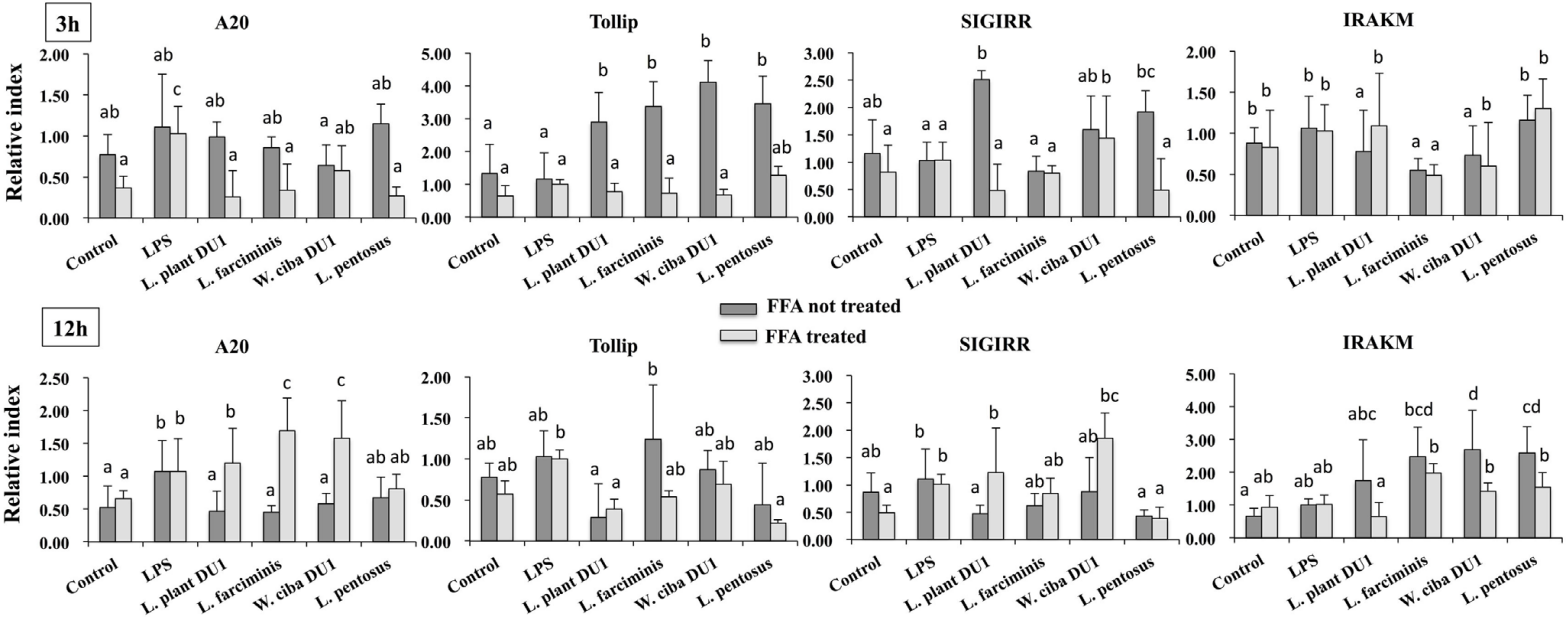
Total cellular fluid (TCF) of lactic acid bacteria (LABs) modulated LPS induced toll-like receptor negative regulators expressions in HepG2 cells. Confluence HepG2 cells were pre-stimulated with LABs TCF (50 µl/ml) for 48 h, then stimulated with LPS for 3 and 12 h. The mRNA levels of A20, SIGIRR, Tollip, and interleukin-1 receptor associated kinase-M1 were determined by RT-PCR. HepG2 cells either with medium or LPS was used as control. Three independent experiments were performed for each case and the mean value was presented as immunomodulatory activity of LABs. Different superscript letters indicate significant differences at the 0.05 levels.

We next analyzed whether TCF of LABs modulate the expression of TLR negative regulators in HepG2 cells stimulated with LPS for 3 and 12 h. Time dependent and cell dependent alternations were observed in the mRNA levels of TLR negative regulators. As shown in Figure 7, stimulation of cells with LPS alone increased the expression of A20, Tollip, SIGIRR, and IRAKM in both FFA treated and not treated HepG2 cells. These LPS induced A20 and IRAKM were diminished when cells pre-stimulated with TCF of all LABs except *L. penstosus* at 3 h. Conversely, the levels of Tollip and SIGIRR were significantly upregulated by TCF of all LAB strains in FFA not treated cells, but not in FFA treated cells. At 12 h, TCF of all LABs were significantly downregulated the mRNA level of A20 when compared to LPS, while significant increases in the level of IRAKM were observed with TCF of all LABs in both FFA treated and not treated HepG2 cells. In addition, stimulation of cells with TCF of LABs except *L. farciminis* decreased the expression of Tollip, while none of the LABs increased the expression of SIGIRR (Figure 7). Conversely, TCF of *L. plantarum* DU1 and *W. cibaria* DU1 showed significant higher mRNA level of SIGIRR in FFA treated cells compared to the LPS.

### Modulation of MAPK and NF-κB Pathways by TCF of LABs in HepG2 Cells

**Figure 8:**
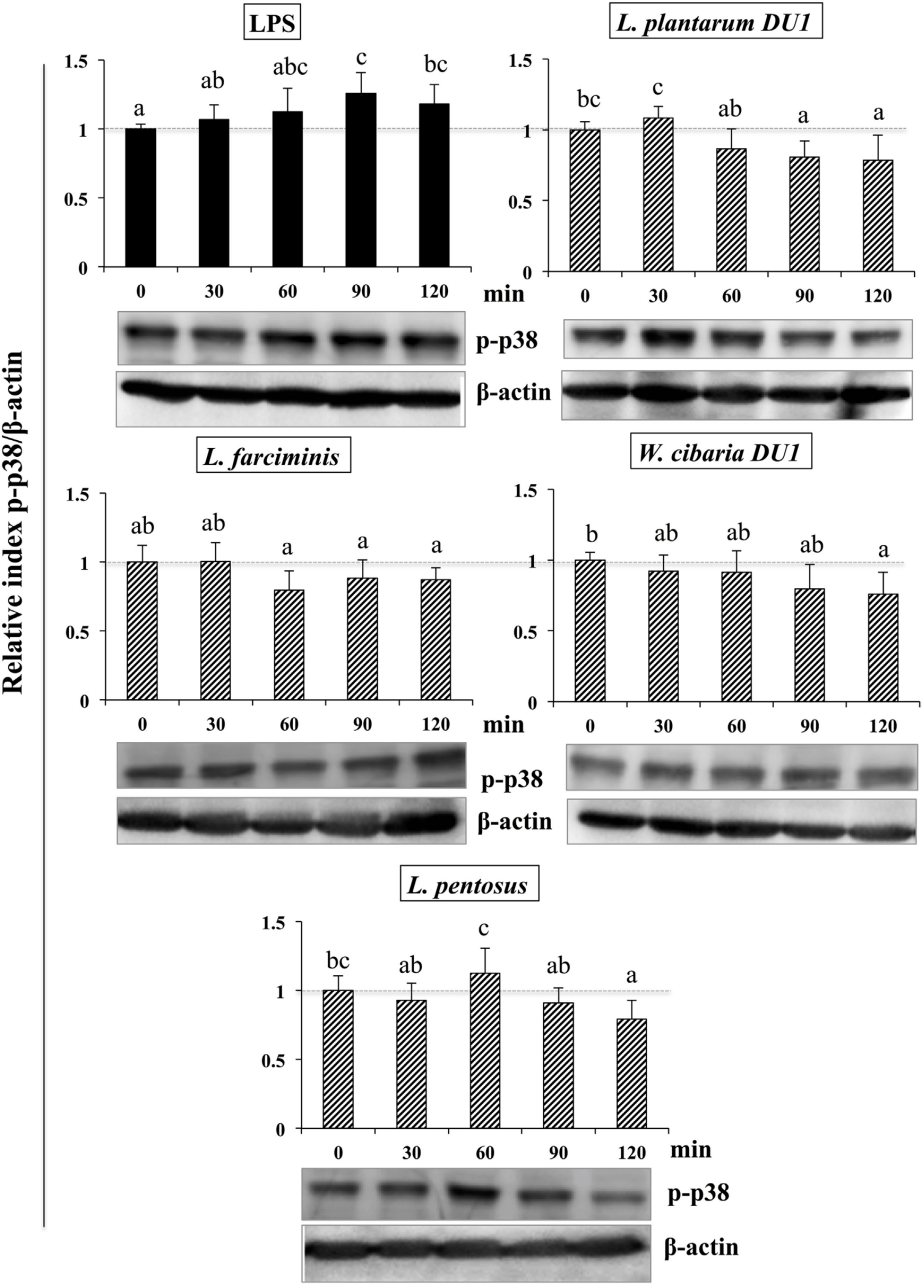
Total cellular fluid (TCF) of lactic acid bacteria (LABs) downregulated expression of p38 mitogen-activated protein kinase (MAPK) in hepatoma HepG2 cells. Confluence HepG2 cells were pre-stimulated with LABs TCF (50 µl/ml) for 48 h, then stimulated with LPS for different time intervals (0, 30, 60, 90, and 120 min). Western blot was performed to determine the phosphorylation of p38 MAPK at the indicated time points. The bar graphs represent the results of three independent experiments. The image J software was used to determine the intensities of proteins bands. Different superscript letters indicate significant differences at the 0.05 level.

It is well known that signaling of TLRs is able to activate MAPKs and NF-κB pathways, which are important for the production of inflammatory and anti-inflammatory cytokines/chemokines and interferons (IFNs). Therefore, we next analyzed whether LAB strains modulated LPS mediated activation of p38 MAPKs and p65 NF-κB pathways in HepG2 cells. To accomplish this, cells were treated with TCF of LABs for 48 h, then with LPS at different time points. Figure 8 that show the phosphorylation of p38 MAPK in HepG2 cells, LPS was able to upregulate the level of p38 MAPK in a time dependent manner. Conversely, cells stimulated with TCF of LABs reduced the LPS induced MAPKs activation by down-regulating the phosphorylation of p38 MAPK. However, TCF of LABs mediated reductions were also varied with time and strain dependent. Stimulation of cells with *L. plantarum* DU1, *L. farciminis*, and *W. cibaria* DU1 decreased the level of p-p38 after 30 min, while strain *L. pentosus* showed reduced levels at 30, 90, and 120 min. These results are remarkably lower as compared to LPS.

**Figure 9:**
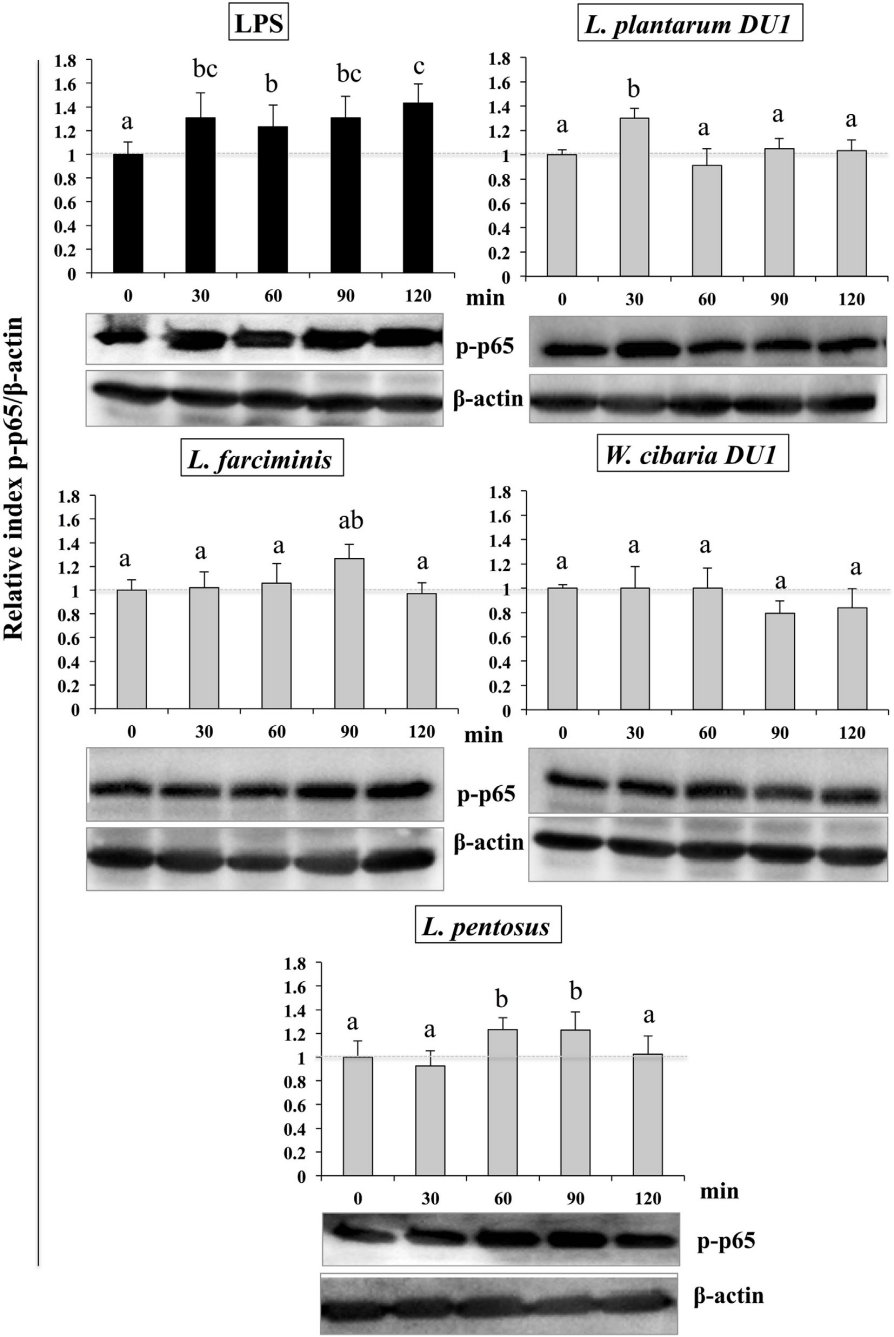
Total cellular fluid (TCF) of lactic acid bacteria (LABs) upregulated expression of p65 NF-κB in hepatoma HepG2 cells. Confluence HepG2 cells were pre-stimulated with LABs total cellular fluid (TCF) (50 µl/ml) for 48 h, then stimulated with LPS for different time intervals (0, 30, 60, 90, and 120 min). Western blot was performed to determine the degradation of p65 NF-κB at the indicated time points. The bar graphs represent the results of three independent experiments. The image J software was used to determine the intensities of protein bands. Different superscript letters indicate significant differences at the 0.05 level.

Similarly, stimulation of HepG2 cells with TCF of LABs downregulated the phosphorylation of p65. The level of p65 NF-κB was also varied with different time points (Figure 9). TCF of *L. plantarum* DU1 significantly augmented the level of p-p65 at 30 min, and followed by decreased at remaining stimulation periods (60–120 min). In addition, HepG2 cells stimulated with *L. farciminis* and *W. cibaria* DU1 downregulated the level of p-p65 after 30 min, while *L. pentosus* showed reduction at 30 and 120 min, but not at 60 and 90 min. These results indicate that MAPKs and NF-κB pathways were transiently modulated when cells were treated with TCF of LABs prior to LPS treatment.

## Discussion

In this study, we demonstrated that TCF of LAB strains reduced the accumulation of lipid and modulated the inflammatory response induced by LPS in hepatoma HepG2 cells. Destruction of intestinal barrier function increased the translocation of gut bacteria, leading to an inflammation and liver injury. Injection of LPS resulted in destruction of gut barrier function, increased bacterial translocation, higher level of proinflammatory cytokine production, and liver injury in mice (22). Probiotics are reported to have several beneficial effects, including amelioration of liver diseases in the host. Moreover, administration of probiotics was found to prevent intestinal damage and liver injury by maintaining the intestinal barrier function and reducing the level of bacterial translocation to the liver (22). In addition, probiotics have been shown to reduce ulcerative colitis and improve colonic barrier integrity, as well as to induce mucosal immunity (23, 24). Another study documented the possible role of probiotics *in vivo*; specifically, the ability of probiotic LGG to attenuate liver steatosis and liver injury *via* maintenance of gut barrier integrity and amelioration of endotoximia (20). The probiotic mediated improvement of intestinal barrier function and prevention of hepatic disease may be due to the secretion of anti-inflammatory mediators, promotion of tight junction formation, and maintenance of epithelial cell integrity (25, 26). Probiotics VSL#3 diminished the production of colonic TNF-α and IFN-γ and hepatic IL-6 *via* suppression of NF-κB pathway (22, 27). However, the beneficial effects of probiotics vary with type and quantity of strains (28). Therefore, it is very important to screen for novel strains with probiotic potential to restore intestinal homeostasis and attenuate liver inflammatory responses and injury. Many studies have been attempted to analyze the beneficial effects of probiotics on amelioration of liver disease *in vivo*; but there have been very few studies *in vitro*. HS is associated with increased accumulation of lipid droplets in the liver cells. Therefore, a measurable *in vitro* steatosis model would increase our understanding of pathogenesis of liver disease and facilitate identification of novel therapeutic agents. Several studies have used oleic acid induced steatosis in HepG2 cells as an effective *in vitro* model to investigate the pathogenesis of fatty liver diseases (29). The present study showed that incubation of HepG2 cells with FFA induced steatosis, which was confirmed by Oil Red O staining and measurement of the optical density. Moreover, this FFA induced HS was reduced when cells were treated with LAB strains, Among which, *L. plantarum* DU1 and *W. cibaria* DU1 showed better reduction as compared to other LAB strains.

Pro-inflammatory mediators and cytokines have been reported to induce pathogenesis of liver diseases (20), and it is well known that LPS is a potent inducer of pro-inflammatory response in liver cells (3, 4). HepG2 cells were highly responded to LPS and exhibited marked increased level of IL-6, IL-1, TGF-β, and TNF-α and intracellular and extracellular nitric oxide (NOs) productions *in vitro* (30–32). Here, we found that HepG2 cells mounted a robust inflammatory response to LPS by expressing exacerbated form of pro-inflammatory cytokines such as IL-6, CXCL8, CCL2, and TNF-α. Our results are consistent with results of other study (30, 33). The effect of these inflammatory cytokines has been shown to induce production of several inflammatory mediators and create complex networks of genes interactions in liver cells, leading to the initiation of several inflammatory signaling cascades (34). Therefore, it is very important to regulate LPS induced inflammatory signaling *in vitro*. Intriguingly, stimulation of HepG2 cells with TCF of LAB strains attenuated LPS induced pro-inflammatory response in a time dependent manner. LAB strains were started to reduce LPS mediated cytokine/chemokine production at 3 h, while marked effects were observed at 12 h for CXCL8 and TNF-α. Among the selected LAB strains, TCF of *L. plantarum* DU1 and *W. cibaria* DU1 led to great decreases in IL-6, CXCL8, CCL2, and TNF-α expression. Conversely, TCF of *L. pentosus* was failed to reduce the level of IL-6 and CCL2 expression at 12 h, while it led to significant reductions in the mRNA level of CXCL8 and TNF-α in HepG2 cells that were not treated with FFA. Conversely, LAB strains exhibited different patterns of inhibitory activity in FFA treated HepG2 cells.

We next analyzed the immunomodulatory activity of LAB strains using an *in vitro* co-culture system. Briefly, HepG2 cells were stimulated with heat-killed LAB strains, which indirectly activated the THP-1 cells to respond to LPS by inducing HepG2 cells to produce several soluble factors. TNF-α is one of the prime pro-inflammatory mediators of inflammation and immune response. The increased TNF-α production is believed to implicate tissue injury and to induce apoptosis (35). Furthermore, TNF-α is considered to be a crucial factor for the development and progression of NAFLD and steatohepatitis through the upregulation of molecules related to inflammatory cytokines, lipid metabolism, and liver fibrosis (36). The level of TNF-α has been shown to be significantly higher in alcoholic patients (37). Moreover, mice lacking tumor necrosis factor receptor type 1 showed inhibition of liver fibrosis induced by carbon tetrachloride (38). In the present study, THP-1 cells were found to be highly responsive to LPS and to secrete higher amounts of TNF-α. However, LPS induced TNF-α production was decreased when THP-1 cells were co-cultured with HepG2 cells that had been stimulated with heat-killed LAB strains, and treatment with all LAB strains led to better reduction compared to LPS. These findings are similar to those of a previous study in which probiotic LGG treatment ameliorated alcohol induced liver inflammation by decreasing the production of TNF-α *in vivo* (30).

We also analyzed the expression of IL-10 and TGF-β in THP-1 cells. IL-10 is a prime immunoregulatory cytokine that plays a crucial role in amelioration of chronic liver diseases, and it production has been observed in hepatocytes, KC, HSC, and liver associated lymphocytes (39). Moreover, many studies have shown an anti-fibrogenesis role of IL-10. For example, mice deficient with IL-10 developed more fibrosis compared to control mice, indicating a beneficial role of IL-10 in the progression of fibrosis induced by CCL4 in mice (40). In contrast to IL-10, the signaling of LPS/TLR4 in hepatic stellate cells plays a crucial role in the progression of liver fibrosis by inducing the expression of cytokines/chemokines that recruit KC cells, which secrete the profibrogenic cytokine TGF-β (3). In addition, TGF-β/Samds signaling is known to suppress the development of tumor (41), whereas Samds independent TGF-β signaling drives tumor progression (42). A previous *in vitro* study also showed that treatment of HepG2 cells with LPS increased the expression of TGF-β at the mRNA and protein level (33). In our study, HepG2 cells treated with LAB strains significantly increased the mRNA level of IL-10 in THP-1 cells, while the level of TGF-β was decreased, but not significantly, by *W. cibaria* DU1 and *L. pentosus*. Additionally, VSL#3 probiotics were able to protect mice against MCD induced liver fibrosis through the inhibition of TGF-β signaling and its expression *in vivo* (43). Moreover, the expression of IL-6 was diminished by all strains except *L. pentosus*, whereas decreased levels of IL-1β were observed with *W. cibaria* DU1, *L. farciminis*, and *L. pentosus*. IL-1β is a key pro-inflammatory cytokine that plays a promising role in the progression of several liver diseases (44). The reduced level of liver enzymes, hepatic injury, apoptotic hepatocytes, IkB, and TNF-α were mounted in IL-1β knockout mice (44). Finally, the expression of TLR2 and TLR4 at mRNA level were also analyzed in THP-1 cells. There was no marked change observed in the mRNA level of TLR2, whereas cells stimulated with all LABs significantly increased the expression of TLR4. These results are consistent with those of another study (44).

**Figure 10:**
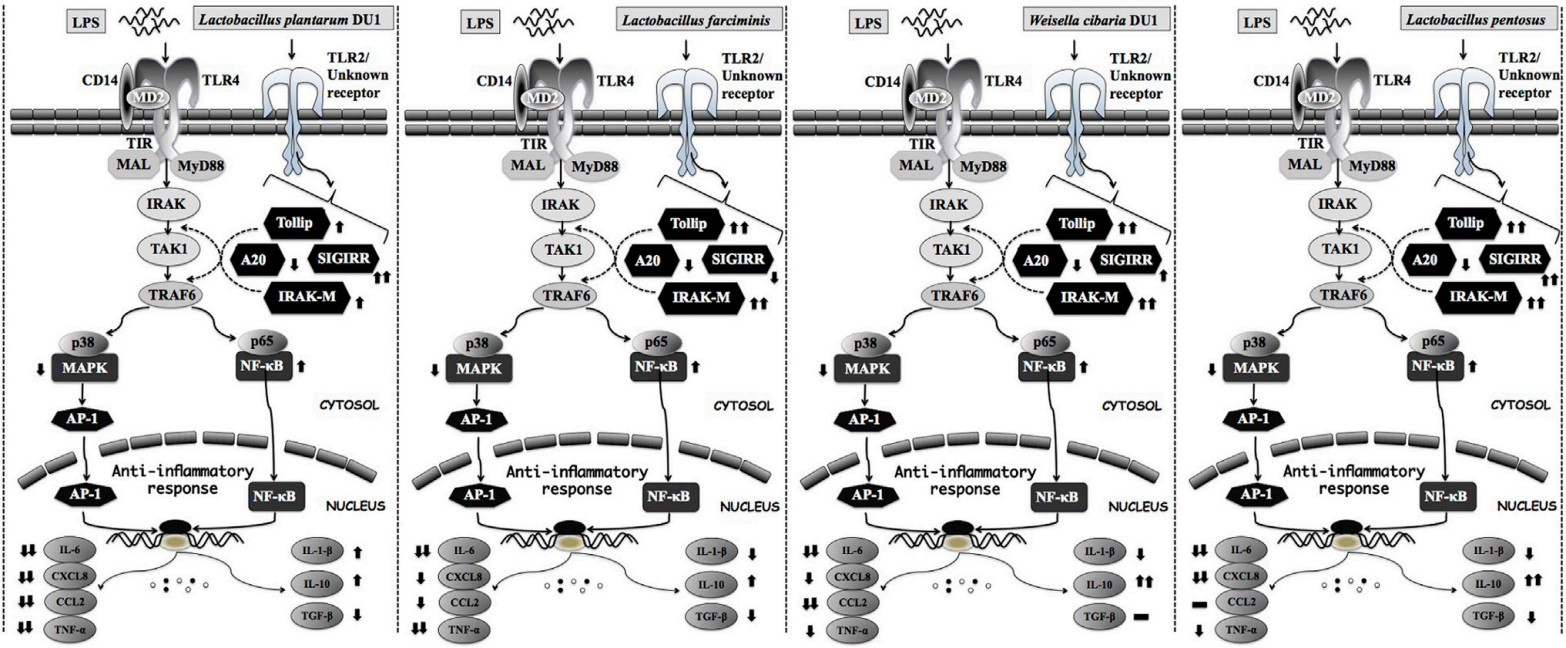
Proposed model for the mechanisms behind the immunomodulatory activity of different lactic acid bacteria strains on HepG2 and THP-1 cells under LPS induced inflammatory state.

Toll-like receptor is a primary sensor for PAMPs that regulates the innate and adaptive immune responses against invaders. However, it is activation proceeds signaling neither beneficial nor pathogenesis (44). TLR over activation is mostly proceeds signaling to pathogenesis of several diseases. Several negative regulatory proteins (SIGIRR, A20, Tollip, Bcl3, SOCS1, and IRAKM) are believed to control or regulate the overactivation of TLRs and to maintain host immunological balance (45, 46). Therefore, in this study, we examined the expression of A20, Tollip SIGIRR, and IRAKM in HepG2 cells. LPS induced FFA treated and un-treated HepG2 cells to express higher levels of A20, Tollip, SIGIRR, and IRAKM. A20 is a zinc finger protein that can repress TNF-α induced NF-κB activation (47). Mice deficient in A20 had more inflammation and hyper-responsiveness to LPS (48). SIGIRR inhibits TLR4 signaling by competing with transcription factors and adaptor proteins for binding sites. IRAKM exhibits different mechanisms to regulate TLR4 signaling, which prevents dissociation of IRAKs from MyD88 by inhibiting TLR4/MyD88 mediated signaling (48). Cells treated with TCF of LABs modulated LPS induced negative regulator expression in both FFA treated and untreated HepG2 cells. At 3 h, TCF of LABs decreased A20, while Tollip and SIGIRR were increased (except TCF of *L. farciminis*) in untreated HepG2 cells. Conversely, IRAKM was increased, while A20 and SIGIRR were decreased in cells treated with all LABs at 12 h. However, TCF of LAB exhibited remarkable differences in modulation patterns in FFA treated HepG2 cells. The inflammatory and anti-inflammatory cytokine production were mainly through TLRs mediated MAPKs and NF-κB activations. MAP kinase was able to regulate cell proliferation, differentiation, morphogenesis, inflammation, and apoptosis by responding to signals that originated from cytokines, hormones, and growth factors (49). NF-κB is a mediator of inflammatory signaling, and its activation induces downstream the targeted genes to regulate cell survival, cell proliferation and migration, and innate and adaptive immune responses (50). Lipopolysaccharide is a known inducer of both MAPKs and NF-κB activities; therefore, we investigated whether TCF of LABs modulates LPS induced p38 MAPK and p65 NF-κB activation in HepG2 cells. Both p38 MAPK and p65 NF-κB pathways are crucial for cytokine production by splenic cells of mice that have been stimulated with *L. casei* (51). We found that TCF of LABs attenuated LPS induced inflammatory response in HepG2 cells by down-regulating the phosphorylation of p38 MAPK and p65 NF-κB proteins (Figure 10).

In conclusion, the results of this study indicate that TCFs of LABs reduced HS and attenuated LPS induced inflammatory response in HepG2 cells. Molecular mechanisms involved in the beneficial activity of LABs including suppression of cells to express decreased levels of inflammatory cytokines/chemokine by modulation of expression of negative regulators of TLR, as well as the p38 MAP kinase and p65 NF-κB pathways. In addition, heat-killed LABs were able to suppress LPS induced inflammatory response in THP-1 cells indirectly through the induction of HepG2 cells to produce more soluble factors. The results of this study increase our understanding of molecular mechanisms through which LAB strains dampen HS and mitigate LPS induced inflammatory response in both HepG2 and TPH-1 cells. Collectively, the data presented herein indicate that LABs exert profound immunoregulatory effects on liver cells that are hypo-responsive to LPS. However, animal and clinical trials are needed to corroborate the results of this study.

## Author Contributions

PK and HK designed the work, discussed the experiment and results. PK and HK performed the assays and wrote the manuscript. All authors read and approved the manuscript for submission.

## Conflict of Interest Statement

The authors declare that the research was conducted in the absence of any commercial or financial relationships that could be construed as a potential conflict of interest.
